# Magnetoelectric Extracellular Vesicle Latency-Targeting (MELT) Nanotherapeutic for the Block-Lock-and-Kill HIV Eradication Strategy

**DOI:** 10.3390/biomedicines13010147

**Published:** 2025-01-09

**Authors:** Mickensone Andre, Nagesh Kolishetti, Adriana Yndart, Arti Vashist, Madhavan Nair, Andrea D. Raymond

**Affiliations:** 1 Herbert Wertheim College of Medicine, Cellular and Molecular Medicine, Florida International University, Miami, FL 33199, USA; mandre@mit.edu (M.A.); ayndarta@fiu.edu (A.Y.); avashist@fiu.edu (A.V.); nairm@fiu.edu (M.N.); 2 Institute of Neuroimmune Pharmacology, Florida International University, Miami, FL 33199, USA; 3 Biomolecular Sciences Institute, Florida International University, Miami, FL 33199, USA

**Keywords:** extracellular vesicles, HIV latency, MENPs, microglia, MELT

## Abstract

Background: Human immunodeficiency virus (HIV) establishes latent infections in cellular reservoirs, including microglia. HC69 cells, a microglial model of HIV latency, contain an HIV promoter long terminal repeat (LTR)-GFP reporter and were used for testing the efficacy of a two-step magnetoelectric nanoparticle (MENP) and extracellular vesicle (xEV) latency-targeting (MELT) nanotherapeutic. GFP expression in HC69 at rest is low (GFP^Lo^), and upon exposure to LTR, transcription-activating agents (i.e., TNF-α) are induced to be high expressing (GFP^Hi^). Methods: The first step of MELT utilized ZL0580, an HIV Tat inhibitor loaded into EVs (80%) via incubation. ZL0580-EVs were taken up by GFP^Lo^ and blocked LTR transcriptional reactivation by 50% and were 90% less toxic than ZL0580 alone. The second step in MELT involved conjugation of monomethyl auristatin E (MMAE) to MENPs. HPLC measurements showed 80% MMAE attachment to MENPs. Flow cytometry-based measurements of the membrane potential indicated that the membranes of GFP^Hi^ HC69 were 60% more polarized than GFP^Lo^ HC69 cells. More MMAE–MENPs were internalized by GFP^Lo^ HC69. Results: Using a mixed-cell blood–brain barrier (BBB) Transwell model, we demonstrated that 20% of MELT crossed the BBB, was taken up by HC69 cells, and reduced LTR reactivation by 10%. Conclusions: Overall, this study demonstrated that MELT can potentially be utilized as a nanotherapeutic to target HIV latency in microglia.

## 1. Introduction

Microglia are the major cellular reservoir of latent HIV in the central nervous system (CNS), and these cells contribute to chronic neuroinflammation and neurological diseases, such as HIV-associated neurocognitive disorder (HAND) [1,2,3]. In 2020, an estimated 50% of people with HIV experienced HAND despite successful anti-retroviral (ARV) drug therapy [4]. ARVs target active HIV infection. However, HIV proviruses (defective or replication-competent) can remain active in cellular reservoirs like microglia and produce viral proteins and mRNAs that are detrimental to neuronal health [5]. Moreover, some ARVs have low CNS penetrance, with reduced capacity to bypass the blood–brain barrier (BBB) [3,6]. Therefore, new drugs are needed to bypass these anatomical barriers, gain access to the CNS, and target latently infected cells to stop HIV transcription.

There are several experimental strategies to eradicate HIV. The shock-and-kill approach focuses on reactivating latent reservoirs with latency-reversing agents (LRAs) and then eliminating the infected cells through exposure to ARVs and other antiviral agents [3,7]. However, this strategy has several limitations, such as no specificity to target only infected cells and viral immune escape due to viral mutation [8]. In addition, the process of reactivating HIV reservoirs can expose and infect other non-infected cells or only reactivate a small subset of HIV-infected cells. Moreover, eliminating infected cells is challenging because cell targeting is inaccurate, and the immune cells, such as the CD8 T cells, are exhausted and unable to kill HIV-infected cells [9]. The block-and-lock eradication strategy is like the shock-and-kill strategy. However, block-and-lock focuses on blocking reactivation and preventing the proviruses from becoming transcriptionally active [10,11]. This strategy also needs more specificity, and components within block-and-lock have difficulty crossing the BBB [12].

The experimental goal of this project merges aspects of the block-lock-and-kill strategy and improves the specificity of the treatment to suppress the HIV provirus. Here, we show preliminary data in which the block-lock-and-kill strategy implemented with exosomal extracellular vesicles (xEVs) and magnetoelectric nanoparticles targets microglia latently infected with HIV and penetration across the BBB. We developed and tested the efficacy of a magnetoelectric exosomal EV latency-targeting (MELT) nanotherapeutic to block the transcriptional reactivation of HIV provirus with ZL0580, a highly toxic HIV-Tat inhibitor [13], encapsulated in xEVs, and then magnetoelectric nanoparticles (MENPs) loaded with monomethyl auristatin E (MMAE) to eliminate HIV-infected cells. The microglia cell line HC69 has a GFP reporter construct that models HIV latency [14]. When the HIV provirus is inactive in the HC69 cells (latent, GFP^Lo^), little to no GFP is produced. However, when transcriptionally reactivated, the HC69 cells will produce elevated levels of GFP. The parental cell line for HC69, C20 cells, was used as the uninfected control. Findings show that in MELT, the Tat inhibitor was significantly less toxic and that the MENP–MMAE part of MELT selectively killed the GFP^Hi^ HC69 cells. These findings suggest that the MELT nanotherapeutic could be a novel strategy for HIV eradication in the CNS using the block-lock-and-kill approach.

## 2. Materials and Methods

### 2.1. Cell Culture

HEK293T cells were cultured in DMEM media (Gibco, cat#11965092, Thermo Fisher Scientific, Miami, FL, USA), supplemented with 10% FBS (Gibco, cat#A5670801, Thermo Fisher Scientific, Miami, FL, USA) and 1% penicillin and streptomycin, at 37 °C with 5% CO_2_. HC69 cells were cultured in DMEM/F12 media (cat#SH30023.02, Gibco, Thermo Fisher Scientific, Miami, FL, USA) supplemented with 1% FBS (cat#A5670801, Thermo Fisher Scientific, Miami, FL, USA) and 1% penicillin-streptomycin (cat#151408, Thermo Fisher Scientific, Miami, FL, USA), 100 ug/mL of normocin (ant-nr-05, InvivoGen, San Diego, CA, USA) and 1 μM of dexamethasone (cat#50-02-2, Millipore Sigma, Burlington, MA, USA) at 37 °C, 5% CO_2_. C20 cells were cultured in DMEM medium supplemented with 10% FBS and 1% penicillin-streptomycin. All cells were seeded at 2 × 10^5^ cells/mL in a T-75 flask, and the medium changed every three to four days.

### 2.2. HIV Infection

C20 cells were cultured in DMEM supplemented with 10% FBS and 1% penicillin-streptomycin were seeded into a 6-well plate at 3 × 10^5^ cells per well and allowed to adhere overnight, and incubated at 37 °C, 5%CO_2_. Polybrene (cat#TR-1003, 8 μg/mL, MilliporeSigma, Burlington, MA, USA) was then added to the cells and the cells were incubated for 2 h at 37 °C, 5%CO_2_. The cells were then infected with NLAD8 HIV (R5-Tropic) at a MOI of 1 and incubated for 2 h at 37 °C, 5%CO_2_. After infection, the supernatants were removed, the adherent cells were washed with PBS (1 mL of per well), and new growth medium was added. Seven days after infection (dpi), the supernatants were collected and HIV replication was measured using the HIV-1 Gag p24 ELISA (cat# DHP240B, Quantikine ELISA, R&D Systems, Minneapolis, MN, USA).

### 2.3. Membrane Potential and Cell Viability (HIV-Infected vs. Uninfected)

**Figure 1:**
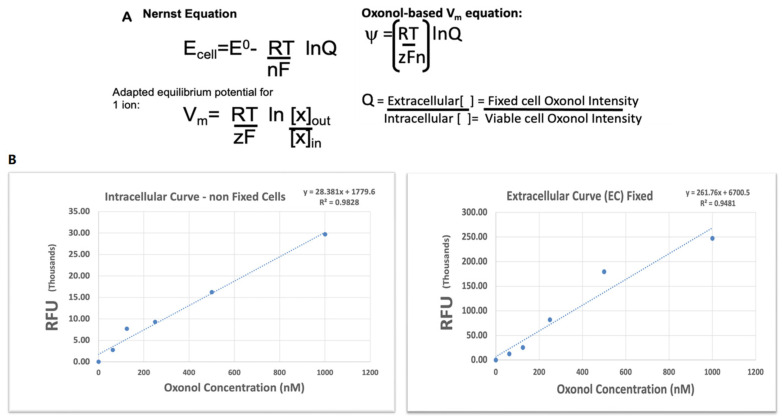
Oxonol fluorescence intensity correlates with concentration. (**A**) Nernst equation (https://physiologyweb.com/, accessed on 7 July 2024) adapted for membrane potential psi. E_Cell_ = cell potential, E^0^ = cell potential under standard conditions; Q = reaction quotient; F = Faraday’s constant (96,485 c/mol); T = temperature (in Kelvin); R = universal gas constant (8.314 J/Kmol; n = number of electrons transferred in the reaction). Adapted equation: ψ = membrane potential, z = ion number; =−1. (**B**) A standard curve was developed by oxonol concentration vs. the RFU values of oxonol intensity excited and detected at FL1 488/520 nm em/ex.

The membrane potential of C20, HC69, and HIV-infected cells was measured via flow cytometry analysis based on a modified Krasznai et al. 1995 protocol. Briefly, the cells were harvested, washed three times with PBS, and then resuspended into two tubes. The cells were centrifuged, and the cell pellets were resuspended in either 70% methanol diluted in PBS (to fix the cells) or PBS alone and then incubated on ice for 30 min. Fixed cells were considered dead, while non-fixed cells were considered viable. Following the incubation on ice, solutions of oxonol III, DiBAC4(3) (bis-(1,3-dibutylbarbituric acid) trimethine oxonol (cat#B413, ThermoFisher Scientific, Miami, FL, USA), or oxonol V, DiBAC4(5) [bis-(1,3-dibutylbarbituric acid) pentamethine oxonol,(cat#B438, ThermoFisher Scientific, Miami, FL, USA) were prepared and added to the cells. Since the HC69 cells held a GFP reporter (488/520 ex/em), oxonol V (590/615 ex/em) was used to measure the membrane potential of both C20 and HC69 cells for accurate comparison. Oxonol III (488/520 ex/em) was used to measure how active NLAD8 HIV infection affects the C20 membrane potential. Fixed and viable cell populations were combined in 200 μL of PBS. From this mixture, 50 μL of the cells were placed into either a solution with 200 μM of oxonol or just PBS. Membrane potential was determined using an adaptation of the Nernst equation (Figure 1). Viability of each cell type—C20, HC69, and HIV-infected C20—was determined using far-red live and dead viability dye (ThermoFisher Scientific, cat# L10120) according to the manufacturer’s instructions. Briefly, the viability dye was added at a ratio of 1:1000 to each cell solution, incubated for 30 min at room temperature, and then analyzed via flow cytometer (BD FACSymphony™ A3, BD Biosciences, Miami, FL, USA).

### 2.4. ZL0580 and MMAE Toxicity and Dose Response

ZL0580 (cat#T13974, Targetmol/Fisher Scientific, Miami, FL, USA) and Monomethyl auristatin E (MMAE)(cat#50-136-6373, Fisher Scientific, Miami, FL, USA) dose–response and toxicity were determined on HC69 cells seeded into a 24-well plate at 5 × 10^4^ cells/mL concentration. Cells were allowed to acclimate at 37 °C, 5% CO_2_ for 48 h and then treated with increasing concentrations of ZL0580 (0.1 μM, 1 μM, 5 μM, 10 μM) in the presence or absence of TNF-α at 300 pg/mL for 24 h. Cells were then harvested and washed three times in cold PBS before staining with far-red live and dead dye for 30 min and then analyzed with a BD Accuri C6 Plus flow cytometer (BD Biosciences, Miami, FL, USA). Cells were allowed to acclimate prior to MMAE treatment as described above for ZL0580. The cells were exposed to MMAE concentrations (0.1, 1, 10, 100, 1000 nM) for 24 h. The cells were harvested, washed three times with cold PBS, and stained with far-red live and dead dye for 30 min as described above for the ZL0580 dose response. Cells were then analyzed with a BD Accuri C6 Plus flow cytometer (BD Biosciences, Miami, FL, USA).

### 2.5. Extracellular Vesicle (EV) Isolation

Extracellular vesicle isolation was performed on culture media derived from the human embryonic kidney (HEK) 293T cell line (ATCC #CRL-3216) and was used as the source of extracellular vesicles (EVs) in the experiments. HEK 293T cells were seeded into four T-75 flasks (10 mL per flask) at a concentration of 2 × 10^6^ cells/mL. After three days, 40 mL of media were collected and pooled. Exosomal EVs were isolated via the differential centrifugation (DC) method. Briefly, the cultured media was centrifuged at 300× *g* for ten minutes to remove cells; the supernatant was then collected and centrifuged at 3000× *g* for ten minutes to remove dead cells or large cell debris. The pellet was discarded, and the 30,000× *g* supernatant was centrifuged at 20,000× *g* for 30 min to remove smaller cell debris, such as organelles and microvesicles. Finally, the 20,000× *g* supernatant was centrifuged at 100,000× *g* for 70 min at 4 °C. The 100,000× *g* exosomal EV pellet was resuspended in PBS and stored at −80 °C for later analysis and drug loading.

### 2.6. ZL0580 Encapsulation in Extracellular Vesicles (ZLExos)

HEK 293T-derived extracellular vesicles (~1 mL) were isolated from tissue culture media via UC, resuspended in 100 μL of ZL0580 (3 mM), and incubated for 30 min at 37 °C. After incubation, the suspension was centrifuged at 100,000× *g* for 120 min at 4 °C. The supernatant was collected to indirectly find the concentration of ZL0580 encapsulated within the extracellular vesicles using HPLC. The pellet was then resuspended in 500 μL of PBS.

### 2.7. SDS/PAGE and Western Blot

Sodium dodecyl sulfate/polyacrylamide gel electrophoresis (SDS/PAGE) was used to analyze extracellular vesicles lysates. Briefly, mini-Protean-TGX AnyKD gels were assembled in the gel electrophoresis cell, running buffer was prepared according to the manufacturer’s instructions, and 15 μg of extracellular vesicles lysate protein per sample prepared in reducing sample buffer were loaded onto the gel. Electrophoresis running conditions were 100 volts for 1 h. After electrophoresis, the gel was removed from the cassette and then placed in a Western blot apparatus, sandwiched between an immunoblot-PVDF membrane, filter paper, and sponges. The apparatus was placed in transfer buffer and electrophoresed at 100 volts for 1 h to transfer proteins to the PVDF membrane. The membrane was blocked with 5% milk in tris-buffered saline with 1% tween-20 (TBST) buffer. Primary antibodies were rabbit anti-ALIX (1:2800), rabbit anti-CD63 (1:2800), rabbit anti-HSP70 (1:2800), rabbit anti-TSG101 (1:2800) in TBST with 1% milk, and horseradish peroxidase (HRP)-conjugated secondary antibody (1:100,000) in TBST with 2% milk. Bands were detected via femto chemiluminescence (cat#PIA43840, Fisher Scientific, Miami, FL, USA) using X-ray film or Chemidoc digital imaging.

### 2.8. Hydrodynamic Size and Zeta Potential

The hydrodynamic size and zeta potential of the extracellular vesicles were found using Zetasizer Dynamic Light Scattering (DLS) (Malvern Panalytical Ltd., Malvern, UK). Briefly, samples were diluted 1:100 in PBS for a final volume of 1000 μL, and the solution was placed in a cuvette and measured on a Zetasizer (Malvern Instruments, Malvern UK), with three measurements consisting of at least 25 reads at 15 s intervals (refractive index = 1.331, viscosity = 0.889, temperature = 25 °C).

### 2.9. Nanoparticle Tracking Analysis (NTA)

A NanoSight LM10 (Malvern Panalytical Ltd., Malvern, UK) was used to capture five frames (30 s each) with a background level of 10 for each sample to find the size and concentration of the isolated EVs for nano-tracking analysis. The camera settings were level 10, and the shutter speed was 30. Captured nanoparticles in 3D distribution images were analyzed using NTA software (version 2.3). The extracellular vesicles samples were diluted 1:100 in PBS to make a 1000 μL solution and filtered through a 0.2 μm filter. PBS was used to wash the instrument after each run. Three 30 s videos were recorded for each extracellular vesicles sample with a detection threshold of 5.

### 2.10. Magnetoelectric Nanoparticle (MENP) Synthesis

The three-step process used to synthesize core–shell MENPs, composed of a core of cobalt ferrite (CoFe_2_O_4_) and a shell of barium titanate (BaTiO_3_), was performed as follows: In step 1, CoFe_2_O_4_ nanoparticles were prepared by adding 116 mg of cobalt II nitrate hexahydrate and 320 mg of iron III nitrate nonahydrate to 30 mL of distilled water. The solution was then stirred at 400 rpm and heated up to 70 °C. Next, 400 mg of polyvinylpyrrolidone were added to the container. Sodium hydroxide was added to the solution dropwise until the pH reached about 11. The mixture was heated to 70 °C for 30 min, diluted with 1:1 water/ethanol three times, and dried overnight. Next, in step 2, the BaTiO_3_ shell was added to the core. First, 58 mg of barium carbonate and 200 mg of citric acid were dissolved in 25 mL of deionized water. Then, 2000 mg of citric acid were dissolved in 25 mL of ethanol with 96 μL of titanium (IV) isopropoxide. The two solutions were then mixed at 70 °C at 400 rpm. A total of 200 mg of the core (CoFe_2_O_4_) were added to a 10 mL solution of 50% ethanol in water and then sonicated for 30 min before being added to the BaTiO3 solution. The mixture was sonicated for 2 h and then allowed to dry overnight on a hotplate at 80 °C and 400 rpm. Finally, in step 3, the dried mixture was heated in a furnace (CMF-1100) at 780 °C for 5 h and then allowed to cool down overnight.

### 2.11. MENP Extracellular vesicles Latency-Targeting (MELT) Nanotherapeutic Preparation

#### 2.11.1. Surface Hydroxylation of MENP

The hydroxylation of magnetic iron oxide nanoparticles (MENPs) was performed by mixing 30% hydrogen peroxide (H_2_O_2_) (1 mL) with MENPs (5 mg) in water. Subsequently, the mixture was sonicated and heated to 100 °C for 5 h. After 5 h, the MENPs were washed in distilled water, followed by ethanol, to remove the hydrogen peroxide. The hydroxylated MENP nanoparticles were then allowed to dry overnight.

#### 2.11.2. Coating MENP with Thiol–PEG–Carboxylic Acid

Thiol–PEG–carboxylic acid polymers were attached to the hydroxylated MENP via covalent binding between the thiol groups on the PEG and the hydroxyl groups on the MENP surface. Thiol–PEG–carboxylic acid (5 mg) and hydroxylated MENPs (5 mg) were dissolved in 1 mL of ethanol. The mixture was then sonicated with a probe sonicator for five cycles of 5 min with one 1 min break. After sonication, the mixture was then stored overnight at room temperature. The nanoparticles were washed three times with distilled water and captured by a magnet to remove free unbound thiol–PEG–carboxylic acid.

#### 2.11.3. Conjugation of MMAE to MENPs

MENP–PEG–MMAE was prepared using the EDC/NHS reaction. Briefly, 200 μL of MENP–PEG–carboxylic acid (5 mg/mL) in distilled water were sonicated and then mixed with 50 μL each of EDC (200 mM) and NHS (50 mM). The mixture was sonicated and then allowed to mix for an added 15 min. Next, 10 μL of MMAE (10 mM) were added to the mixture to create a final concentration of 322.58 μM. The solution was probe-sonicated for 1 min three times and incubated overnight. The solution was washed three times in distilled water. Lastly, nanoparticles were removed from the solution via a magnet and stored at −20 °C, and MMAE concentration was determined using HPLC. The measurement was found by subtracting the concentration of free drug in the supernatant from the initial total amount of drugs, dividing the difference by the total amount of drugs and multiplying this value by 100.

### 2.12. ZLExo Staining and Internalization

ZLExo internalization was examined using four groups: (1) ZLExo stained, (2) ZLExo unstained, (3) extracellular vesicles stained, and (4) post-UC ZL0580 (to confirm that no free unbound ZL0580 pelleted down post-UC). The ZLExo (group 1) and exosomal EV (group 3) solutions (500 μL) were stained with deep red fluorescence cell plasma membrane dye (1 μL) preamble (DRPM dye) from Abcam (ab219942, Waltham, MA, USA). The solutions were incubated at 37 °C for 30 min and then centrifuged at 100,000× *g* for 120 min at 4 °C to pellet down the extracellular vesicles and remove any free unbound dye. The supernatant was discarded, and the pellet was gently washed with PBS twice to remove any unbound dye. After resuspending the pellet in 500 μL of PBS, HC69 cells were seeded into a 24-well plate and incubated for 72 h. Each well was treated for 24 h with one of the four groups (50 μL). The cells were harvested, washed three times in PBS, and measured using flow cytometry with Accuri C6. In a different study, C20 cells were seeded at 5 × 10^4^ into a 24-well plate for three days. Next, each well was treated at various times with 50 μL of DRPM-stained extracellular vesicles and harvested together. The staining intensity was measured using flow cytometry at FL4 675/25 nm.

### 2.13. Determination of MENP Internalization

HC69 cells were cultured in a 10 mm dish for 72 h. The MENP solution (5 mg/mL, 500 μL) was adjusted to pH 4 with (1 M) HCl. Oxonol III (100 μL) was added to the MENP solution, and the probe was used to sonicate the solution five times for 3 min intervals to disperse the MENPs with the dye. The nanoparticles were washed three times in PBS to remove any unbound dye by centrifugation at 5000× *g* for 5 min, and the MENPs were resuspended in PBS (500 μL). Stained MENPs (50 μL) were used to treat the cells at different AC magnetic fields (0.5 or 1.75 mT) and at various times (10 or 30 min). The cells were gently washed three times with PBS to remove any free-stained MENPs. The cells were harvested and washed thrice before measuring via flow cytometry using an Accuri C6 instrument (BD Biosciences, Miami, FL, USA).

### 2.14. In Vitro Blood–Brain Barrier (BBB) Transwell Model

The in vitro BBB Transwell model was prepared using a HTS Transwell 24-well permeable support system (cat#07-200-688, Corning/Fisher Scientific, Miami, FL, USA). The basolateral (brain side) of the BBB was formed by layering a mix of human astrocytes (HAs) (cat#1800, Sciencell, Carlsbad, CA, USA) on the under-face of the Transwell membrane (0.2 microns, Falcon) for a total cell number of 5 × 10^5^ cells per well and incubated for up to 2 h. After incubation, the Transwell was placed in a well of the 24-well companion plate (cat#CLS3513, Costar^TM^, Fisher Scientific, Miami, FL, USA) holding complete astrocyte media (cat#1801, Sciencell, Carlsbad, CA, USA). Human brain microvascular endothelial cells (HBMECs) (cat#1000, Sciencell, Carlsbad, CA, USA) were then added to the apical (blood side) of the membrane insert, and the mixed-cell BBB model was cultured at 37 °C for five days before use in MELT efficacy studies. The HC69 cells were cultured separately in the outer chamber of an insert companion 24-well plate, and after 5–7 days, the HA-HBMEC insert was placed in the plate with HC69 cells to generate the mixed-cell BBB Transwell model. MELT nano-formulations were added on the apical side of the BBB Transwell model. The ZLExo drug was added first to the mixed-cell BBB and incubated for 24 h. The MENPs were added to the apical side and pulled down with a magnet for 30 min. An AC magnetic field of 1.8 mT was applied to internalize the MENP into the HC69 cells. After 24 h, the cell viability, GFP intensity, MENP oxonol V intensity, the BBB’s TEER, and BBB permeability were all measured.

### 2.15. Statistical Analysis

All experiments were conducted three times. Data were analyzed and plotted using GraphPad Prism version 10.1.1. One- and two-way analysis of variance (ANOVA) tests were used to determine statistical significance (*p*-value < 0.05) with a post-hoc test (Kruskal–Wallis test or Tukey’s multiple comparisons test). Origin software 2022 was used to normalize the X-ray diffraction (XRD) data from 0 to 100, creating a baseline that was then subtracted.

## 3. Results

### 3.1. Differential Membrane Potential of HIV Latent and Active Microglia Cells

Oxonol V (bis-(1,3-dibutyl barbituric acid) trimethylene axonal), a fluorescent indicator of cell viability, was used to determine membrane potential. The Nernst equation, used in chemical thermodynamics to calculate the reduction potential of a reaction using absolute temperature, electrode potential, and the concentration of chemicals undergoing oxidation/reduction (Figure 1A), was adapted and used to determine whether HIV infection or latency modulated membrane potential. Oxonol intensity was proportional to the dye concentration; the intensity values of the fixed and viable cells replaced the extracellular and intracellular concentrations of the oxonol V dye, respectively (Figure 1B). The quotient of the fixed cell intensity over the viable cell intensity was implemented into the Nernst equation (Figure 1A).

**Figure 2:**
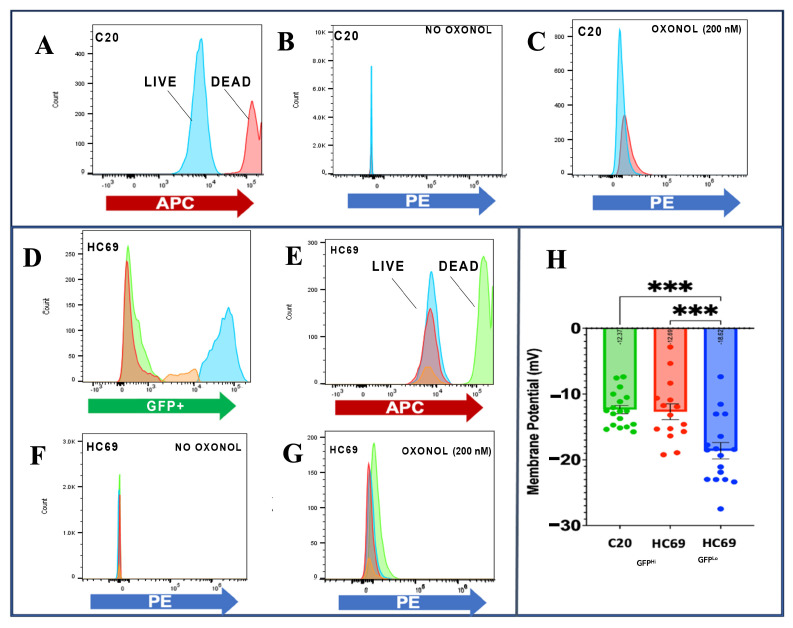
Oxonol-based membrane potential measurement of microglia cell line HC69 and C20 cells. (**A**) Cell viability measured with far-red live and dead dye (APC); blue peaks are the viable cells and red peaks are the fixed/dead cells. (**B**) Fixed and viable C20 cell fluorescence at 0 nM of oxonol (PE). (**C**) Fixed and viable C20 cell fluorescence at 200 nM of oxonol. (**D**) HC69 GFP intensity. Red peaks pertain to GFP^Lo^ cells, blue peaks are GFP^Hi^ cells, and green peaks are fixed cells. (**E**) HC69 cell viability. (**F**) HC69 cell fluorescence at 0 nM of oxonol (PE). (**G**) HC69 cell fluorescence at 200 nM of oxonol (PE). (**H**) Membrane potential of C20, HC69 (GFP^Hi^), and HC69 latent (GFP^Lo^). Statistical significance determined by ANOVA, Dunnett’s test for post hoc analysis, *** *p* > 0.001.

The membrane potential of the HIV latency cell model, the HC69 microglial cell line, was assessed and compared to the uninfected parental cell line C20. The membrane potential of C20 was measured by staining with a far-red dye to separate viable cells (blue) from the fixed/dead cells (red) (Figure 2A). Post-staining and without oxonol V, the fixed and viable cell intensities were near zero (Figure 2B). However, with oxonol III at 200 nM, the fixed cells absorbed more oxonol III dye than the viable cells (Figure 2C). Next, HC69 cells were gated into different populations based on their GFP intensity. GFP^Lo^ represented HIV latency, while GFP^Hi^ cells showed reactivated HIV (Figure 2D). The latent population had the lowest GFP intensity and was labeled red. The active population had the highest GFP intensity and was labeled blue. Some of the population was transitioning from high to low GFP or vice versa. These cells were labeled orange. The following graph gated the fixed cells (green) from viable cells (Figure 2E). In the absence of oxonol V, all cell populations in the study had fluorescent intensities near zero (Figure 2F). However, in the presence of oxonol V, the fixed cells absorbed more oxonol dye than the viable populations (Figure 2G). The membrane potential of the C20 cells, HC69 active (GFPHi) (A), and HC69 latent GFP^Lo^ (L) were compared, and the GFP^Lo^ HC69 cells had a significantly more hyperpolarized membrane potential than the GFP^Hi^ HC69 cells and uninfected C20 cells (Figure 2H). There was no significant difference between the membrane potential of the GFP^Hi^ HC69 and uninfected C20 cells. These findings show that the membrane potential of these HIV latently infected cells differs from that of uninfected C20 and GFP^Hi^ cells.

**Figure 3:**
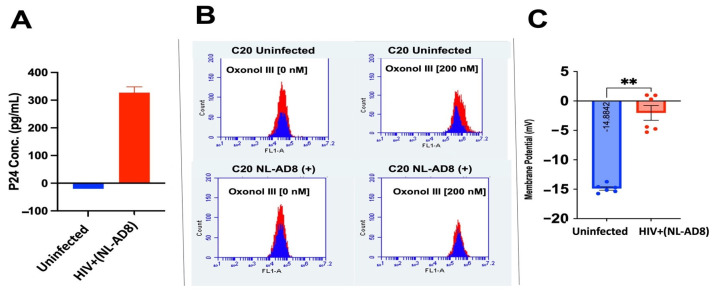
C20 infection with HIV NL-AD8 and membrane potential. (**A**) C20 infection with NL-AD8 was verified by measuring the P24 protein concentration with a p24 ELISA. (**B**) Histogram of uninfected C20 (**top**) and NL-AD8 infected C20 cell (**bottom**) oxonol III intensity detected at FL1 520 nm. Red peaks are fixed cells and blue peaks are viable cells. (**C**) The membrane potential of uninfected vs. infected C20 cells. Statistical significance determined by Student *t*-test, ** *p* > 0.001.

Membrane potential differences between uninfected cells and cells actively infected with HIV were assessed using oxonol III. C20 cells were infected with NLAD-8 HIV and then stained with oxonol III. Infection was confirmed via an HIV-1 Gag p24 ELISA, and the p24 antigen was detected only in HIV-infected C20 cells (Figure 3A). The membrane potential of infected and uninfected C20 cells was measured. Dead/fixed C20 cells were red, and viable C20 cells were blue (Figure 3B). Both infected and uninfected C20 cells had similar background fluorescence intensity when oxonol III was not added. However, when oxonol III was added, the fixed cells absorbed more of the dye than the viable cells. In addition, the dead/fixed and viable cells in the infected cells had oxonol intensity within close range, while within the uninfected cell population, the dead/fixed and viable cells had oxonol III values that were more disparate from each other than within the HIV-infected cells. Overall, the membrane potential of the HIV-infected C20 cells was significantly more depolarized than the uninfected C20 cells (Figure 3C). This finding shows that HIV infection does alter the cell membrane potential.

### 3.2. Cytotoxicity and Dose Response of MELT Components: ZL0580 and MMAE

**Figure 4:**
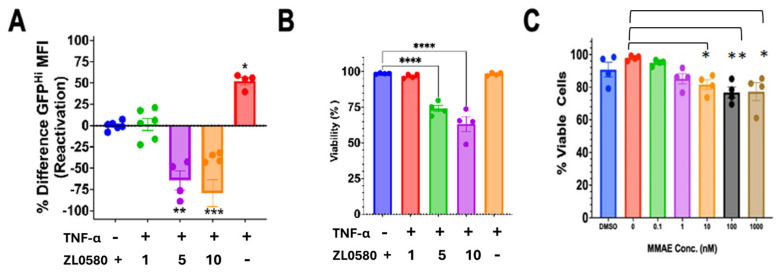
ZL0580 and MMAE cytotoxicity and dose response. (**A**) Flow cytometry analysis of HC69 microglial GFP intensity (FL1) normalized to the NT after 48 h of treatment with ZL0580 and 24 h of treatment with TNF-α. Samples are compared to the untreated sample (ZL0580). (**B**) Flow cytometry analysis of HC69 microglial viability (FL4) after 48 h of treatment with ZL0580 and 24 h with TNF-α treatment. (**C**) Flow cytometry analysis of HC69 viability (FL4) after 24 h of treatment with MMAE. Statistical significance determined by ANOVA, post hoc analysis Dunnett’s * *p* > 0.5, ** *p* > 0.01, *** *p* > 0.001 and **** *p* > 0.0001.

Given that active and latent HIV infection alters the membrane potential of microglia, we explored whether this difference in membrane potential can be exploited for differential treatment of latent and/or actively HIV-infected cells using the MELT nanotherapeutic. MELT consisted of two drugs—ZL0580 and MMAE—loaded on either an organic (exosomal EV (xEV)) or inorganic (MENP) nanoparticle and was used in tandem to block transcriptional reactivation of HIV. Cytotoxicity and dose-dependent responses to MELT components were assessed. The effects of ZL0580, a Tat inhibitor, on HC69 microglial cell viability and HIV reactivation (GFP intensity) were assessed using flow cytometry. The cells were treated with different concentrations of ZL0580 for 24 h and then treated with TNF-α for an additional 24 h. All three concentrations of ZL0580 (1, 5, 10 μM) decreased the GFP intensity (Figure 4A). ZL0580 at 5 and 10 μM significantly decreased the viability of HC69 cells (Figure 4B). Next, we examined the effect of MMAE on HC69 viability. MMAE at 10 nM, 100 nM, and 1000 nM significantly decreased the viability of HC69 cells (Figure 4C). These findings show that these drugs can be toxic at high concentrations.

### 3.3. ZL0580-Loaded Extracellular Vesicles (ZLExo) Characterization

**Figure 5:**
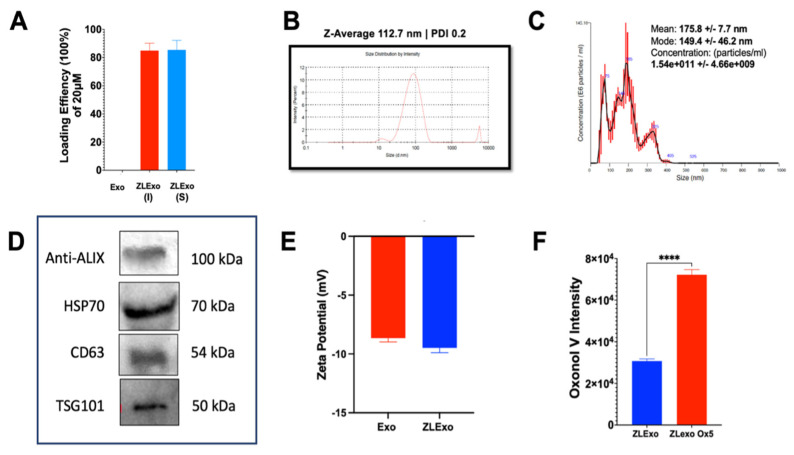
Characterization of extracellular vesicles loaded with ZL0580 (ZLExos). (**A**) Loading efficiency of ZL0580 into extracellular vesicles measured by HPLC. (**B**) ZLExo size and PDI measured by DLS. (**C**) The size and concentration of ZLExos measured by NTA analysis. (**D**) Western blot analysis of exosomal protein markers of ZLExos. (**E**) Zeta potential of ZLExos compared to unloaded extracellular vesicles (Exos). (**F**) Oxonol V fluorescence intensity of ZLExos compared to oxonol V-stained ZLExos. Statistical significance determined by Student *t*-test analysis, **** *p* > 0.0001.

The Tat inhibitor drug (ZL0580) was loaded onto the extracellular vesicles (ZLExos) and characterized. Both sonication and incubation of extracellular vesicles resulted in approximately 80% of ZL0580 encapsulation, indicating there were no significant differences between these methods (Figure 5A). Based on DLS analysis, the average diameter of ZLExos was 112.7 nm (Figure 5B). Next, the average diameter size of the ZLExos based on NTA analysis was 175.8, with most of the ZLExos having a concentration of 1.54 × 10^11^ particles per mL (Figure 5C). Western blot analysis confirmed that the sample has extracellular vesicles expressing exosomal protein markers such as CD63, ALIX, HSP70, and TSG101 (Figure 5D). There were no significant differences between ZLExo and extracellular vesicles zeta potential (Figure 5E). Last, we confirmed that the extracellular vesicles were stained. The fluorescence intensity of ZLExos stained with oxonol V (OX5) was higher than that of the control (Figure 5F). The data confirmed that ZL0580 was loaded into extracellular vesicles.

### 3.4. Characterization of MENP–MMAE

**Figure 6:**
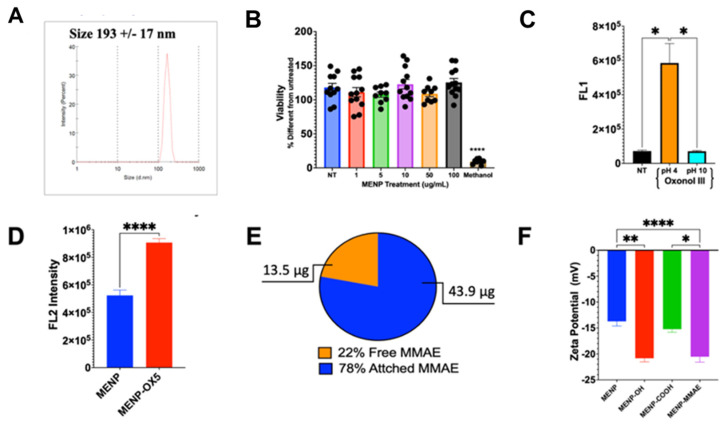
MENP characterization. (**A**) MENP size and PDI measured via DLS. (**B**) XTT analysis of MENP cytotoxicity on HC69 microglial cells. (**C**) MENP fluorescence intensity of unstained MENPs vs. MENPs stained with oxonol III (488/520 ex/em) at different pH. (**D**) MENP fluorescence intensity of unstained MENPs vs. MENPs stained with oxonol V (590/615 ex/em). (**E**) Loading efficiency of MMAE onto MENPs measured via HPLC. (**F**) Zeta potential of MENPs with the different functional groups attached. Statistical significance determined by ANOVA, Dunnett’s test for post hoc analysis, * *p* > 0.5, ** *p* > 0.01, and **** *p* > 0.0001.

After synthesizing the MENPs, the size, cytotoxicity, fluorescence, zeta potential, and drug-loading efficiency of the nanoparticles were characterized. The average hydrodynamic size of the MENPs was 193 nm in PBS (Figure 6A). Next, the MENP nanoparticles at a concentration of 1 to 100 µg/mL were less toxic to the HC69 microglia cells than the non-treated cells (Figure 6B). Two batches of MENPs were stained with either oxonol III or oxonol V to examine the internalization of the nanoparticles into cells. At acidic pH 4, MENPs were stained with oxonol III (Figure 6C). Oxonol V-stained MENP fluorescence intensity was significantly higher than the unstained MENP control (Figure 6D). Next, we attached MMAE to the MENPs and observed a 78% loading efficiency, with about 43.9 μg attached (Figure 6E). To confirm the presence of MMAE on the MENP nanoparticles, we measured the zeta potential of the nanoparticles with different surface functional groups. The zeta potential of MMAE–MENPs was significantly lower than unconjugated MENPs (see Figure 6F). The findings confirm that MMAE can be attached to MENPs.

### 3.5. ZLExo Internalization Effect on HIV Reactivation

**Figure 7:**
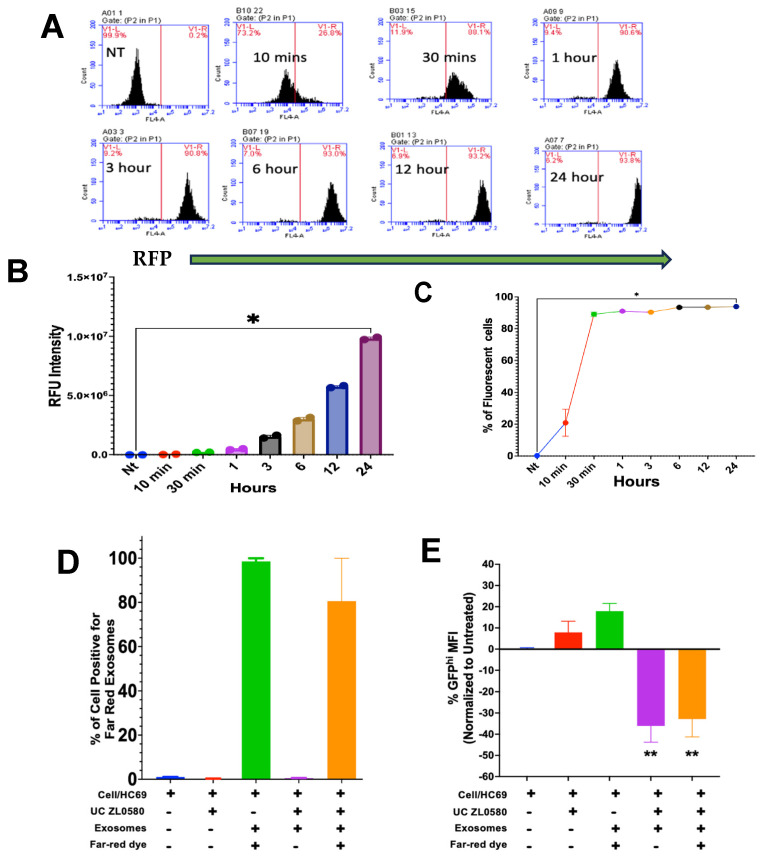
Cellular internalization of ZLExos reduced HIV reactivation. (**A**) Histograms of C20 uptake of RFP-labeled extracellular vesicles at different time points. (**B**) MFI C20 fluorescence intensity post-treatment with far-red (DRPM) extracellular vesicles detected at FL4 660 nm via Accuri C6 flow cytometer. (**C**) Percentages of cells positive for far-red intensity. (**D**) Internalization of stained or unstained ZLExos. (**E**) HIV reactivation of HC69 cells treated with far-red-stained ZLExos, unstained ZLExos, far-red-stained extracellular vesicles, and UC-ZL. Statistical significance determined by ANOVA, Dunnett’s test for post hoc analysis, * *p* > 0.5 and ** *p* > 0.01.

To confirm the uptake of the extracellular vesicles by the microglia cells, the extracellular vesicles were labeled with DRPM dye as described in the Methods section. Within ten minutes, the extracellular vesicles were taken up into the C20 microglial cells (Figure 7A). The highest fluorescence intensity was at 24 h (Figure 7B). Within 30 min, all the cells had taken up the extracellular vesicles, at a rate of 88% (Figure 7C). A time-dependent increase in the fluorescent intensity occurred. A small percentage of cells did not take up the ZLExos. These findings suggest that extracellular vesicles uptake is a slow process that can be impacted by the pharmacokinetics of drugs loaded inside the vesicles.

The internalization of HC69 cells of ZLExos was determined by incubating HC69 cells with ZLExos for 24 h and staining them with a DRPM dye to measure internalization via flow cytometry. The cells containing far-red ZLExos had a high fluorescence (Figure 7D). In terms of HIV reactivation based on the mean fluorescence intensity (MFI) of the GFP^Hi^, the cells treated with ZLExos with or without the dye had a decreased GFP MFI compared to the non-treated cells (Figure 7E). This finding suggests that ZL-0580-EVs can be internalized into the cells and reduce LTR reactivation.

### 3.6. MENP Internalization

**Figure 8:**
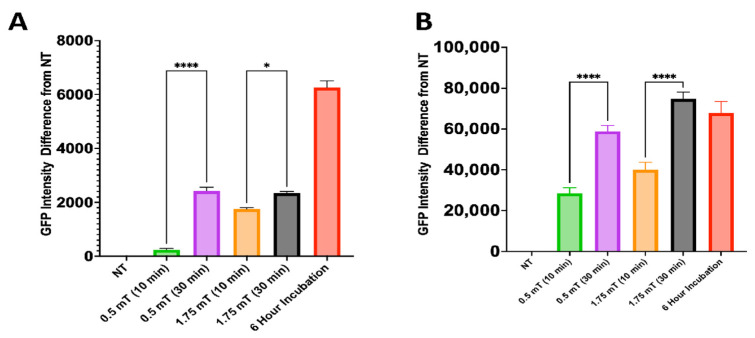
Oxonol III-stained MENP internalization by HC69 cells (GFP^Hi^ and GFP^Lo^): (**A**) GFP^Lo^ population of internalization of oxonol III-stained MENPs under different AC magnetic fields (0.5 mT and1.75 mT) and times (10 min and 30 min). (**B**) GFP^Hi^ population of internalization of oxonol III-stained MENPs under different AC magnetic fields (0.5 mT and 1.75 mT) and times (10 min and 30 min). Statistical significance determined by ANOVA, Dunnett’s test for post hoc analysis, * *p* > 0.5 and **** *p* > 0.0001.

Next, the internalization of MENPs labeled with oxonol III was tested with different magnetic fields (0.5 mT and 1.75 mT) and at various times (10 or 30 min). The cells were also incubated with the nanoparticles for six hours to examine whether the nanoparticles would be naturally internalized. The cells were harvested and then measured via flow cytometry. MENPs incubated with HC69 cells for 6 h had the highest oxonol III intensity among the GFP^Lo^ population (Figure 8A).Within the GFP^Lo^ population, time and the magnetic flux density significantly increased the oxonol intensity associated with the HC69 microglia cells, and about 30 min of incubation in an AC magnetic field of 0.5 and 1.75 mT increased the oxonol III intensity compared to 10 min. Moreover, 10 min of incubation in a 1.75 mT increased the oxonol III intensity compared to 0.5 mT. Within the GFP^Hi^ population, HC69 cells in an AC magnetic field for 30 min at 1.75 mT had the highest oxonol intensity (Figure 8B). Incubation in an AC magnetic field for 30 min in both 0.5 and 1.75 mT had the highest oxonol III intensity compared to 10 min. In addition, the GFP^Hi^ population had the highest oxonol III intensity compared to the GFP^Lo^ population. These observations show that MENPs can enter different cells based on the cell membrane potential.

### 3.7. Effect of Extracellular Vesicles Loaded with ZL0580 (ZLExos) on HIV Reactivation

**Figure 9:**
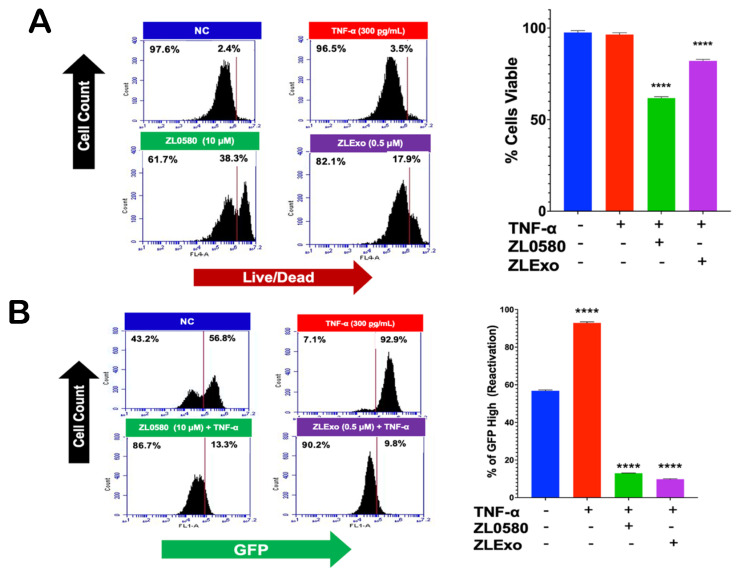
ZLExo effect on HIV latency reactivation. (**A**) Percentage of viable cells treated with ZL0580 and ZLExos. TNF-α was used as a positive control for HIV reactivation. Histograph of GFP intensity after 24 h. (**B**) Percentage of GFP^Hi^ cells reduced when treated with ZL0580 and ZLExos. TNF-α was used as a positive control for HIV reactivation. Samples were analyzed using an Accuri C6 flow cytometer. Statistical significance found by ANOVA, Dunnett’s test for post hoc analysis **** *p* > 0.0001.

HC69 cells were treated with ZLExos for 24 h and then stimulated with TNF-α for an additional 24 h for HIV reactivation. At baseline level (NT), the percentage of cells with high GFP was 56.8% (Figure 9A). However, when treated with just TNF-α, the percentage of cells positive for HIV reaction increased to 92.9%. HIV reactivation decreased to 13.3% in cells treated with ZL0580 alone (Figure 9B), while for the HC69 cells treated with ZLExo (50 μL), HIV reactivation was reduced to 9.8%. The viability of ZLExo-treated HC69 cells was assessed. The untreated and the positive TNF-α control cells had a viability of 97.6% and 96.5% (Figure 9). When treated with the ZL0580 drug alone, the cell viability reduced to 61.7%. When treated with our ZLExo nanoparticles, the cell viability was reduced to 82.1%. These findings confirmed that ZLExos are less toxic to the cells than the drug alone.

### 3.8. MELT BBB Transmigration

**Figure 10:**
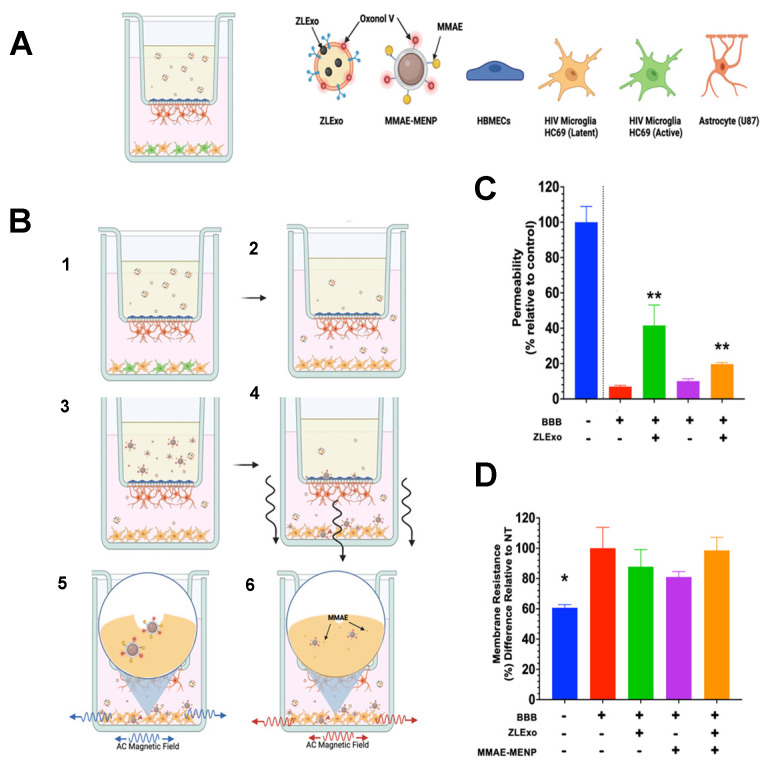
Schematic overview of MELT and its effect on the BBB. (**A**) Mixed-cell in vitro BBB model consisting of primary human astrocytes and brain microvascular endothelial cells forming the BBB on the Transwell membrane. Active or latent HC69 cells were plated in the outer chamber. (**B**) Step 1: (1) ZLExos were added to the inner chamber of the Transwell. (2) After 24 h, some of the ZLExos crossed the BBB and were internalized into the HC69 cells and induced latency. (3) Step 2: After 24 h treatment with ZLExos, MENPs were placed into the inner chamber of the Transwell. Residual ZLExos should still have been in both chambers. (4) A DC magnetic field was placed to attract the MENPs down to the lower/outer chamber and contact the HC69 cells. (**C**) Schematic of MENP internalization and drug release. (5) After the MENPs encountered the HC69 cells, a low AC magnetic field (blue) was generated to stimulate the MENPs to enter the HC69 cells via nanoelectroporation. (6) A higher AC magnetic field (red) was generated to release the MMAE into the cell’s cytosol. (**C**) Permeability and transendothelial membrane resistance (TEER) of the in vitro BBB Transwell model 48 h (about 2 days) post-MELT treatment. (**A**) Permeability. (**D**) TEER was not significantly changed by MELT. Statistical significance found by one-way ANOVA (Kruskal–Wallis test for post hoc analysis), * *p*-value < 0.05, ** *p* = 0.0001, ns—not significant.

Next, the ability of the magnetoelectric exosomal EV latency-targeting (MELT) nano-formulation to cross the blood–brain barrier (BBB) was tested using an in vitro BBB Transwell model consisting of HBMECs and astrocytes on the membrane an

d HC69 cells plated in the outer chamber (Figure 10A). A diagram of our treatment plan is depicted below (Figure 10B). To determine whether MELT disrupted the integrity of the BBB, we measured the permeability and the membrane resistance (TEER) of the BBB. The permeability of our in vitro BBB Transwell model was measured using a dextran–FITC transport assay and detected at 520 nm. ZLExos increased the BBB permeability when treated alone or with MMAE–MENPs (Figure 10C). Surprisingly, MMAE–MENPs did not affect BBB permeability or integrity. TEER values of each group were not significantly different from the no-treatment (NT) group (Figure 10D). These results show that MELT did not significantly alter the BBB integrity.

### 3.9. MELT Effects on HIV Reactivation Post BBB Transmigration

**Figure 11:**
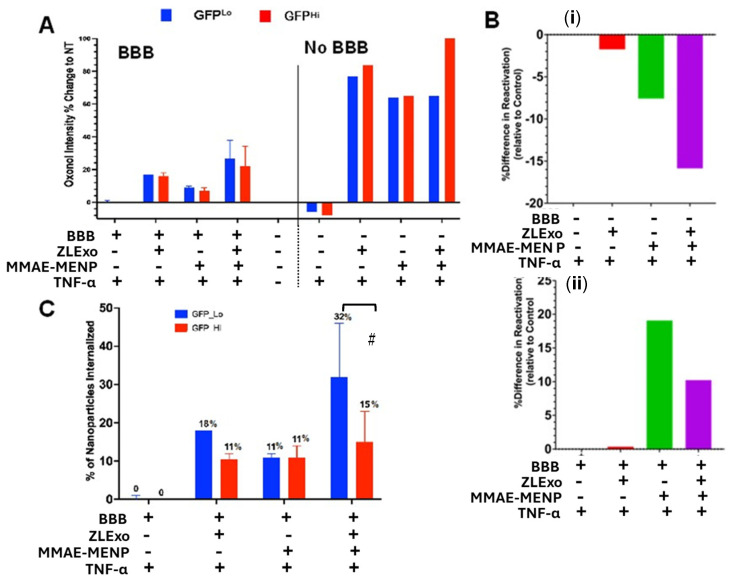
MELT BBB transmigration and efficacy. (**A**) Cells treated with MELT nanoparticles (ZLExos and MMAE–MENPs) in the in vitro BBB Transwell model. HC69 cell viability was assessed with a far-red live/dead dye. The intensity was measured via flow cytometry. (**B**) HC69 cell GFP percentage difference in reactivation after treatment with MELT nanoparticles (ZLExos and MMAE–MENPs) in the in vitro BBB Transwell model. (**C**) Ratio of cells to MENPs comparing BBB to no BBB. # Statistical trend via two-way ANOVA, # *p* < 0.1.

Next, we assessed whether MELT could effectively cross the BBB and be taken up preferentially by latent cells. MELT nanoparticle transmigration across an in vitro BBB Transwell model was evaluated and HC69 internalization was found via flow cytometry. Nanoparticles were stained with oxonol V so as not to interfere with HC69 GFP intensity. As a control, HC69 cells were treated with MELT nanoparticles in the presence and absence of the BBB Transwell. The cells internalized more MMAE–MENP nanoparticles in the absence of the BBB, as expected. The GFP^Hi^ cells in the absence of the BBB model had slightly greater uptake than the GFP^Lo^ cells (Figure 11A). This trend was not seen in the presence of the BBB. The MELT uptakes of the GFP^Lo^ and GFP^Hi^ cells were not significantly different. However, there was a slight trend in which GFPLo cells took up more of the MELT. Comparing the impact of MELT on reactivation post-BBB transmigration showed a 15% relative reduction in reactivation compared to the untreated controls in the absence of the BBB (Figure 11B(i)). In the presence of the BBB, ZLExo alone did not reactivate, while the MMAE–MENPs resulted in an almost 20% increase in reaction compared to the untreated cells. The combination of ZLExos and MMAE–MENPs resulted in only a 10% increase in reactivation (Figure 11B(ii)). GFP^Lo^ cells had two-fold more MELT particles than the GFP^Hi^, as found by flow cytometry (Figure 11C).

Taken together, these preliminary findings show that latent cells can be targeted and that MELT was preferentially taken up by GFP^Lo^ cells, suggesting that MELT can selectively restrict HIV reactivation in latently infected microglia.

## 4. Discussion

These results support earlier reports that HIV infection alters the membrane potential, and this alteration can be exploited by MENPs to target HIV-infected cells, especially between active and latent cells [15,16,17]. MENPs can create an electrical impulse that creates pores on the cell membrane and allows the nanoparticles to enter the cells, like electroporation but on a nano-level [18]. Nanoelectroporation by MENPs can be targeted to specific cells based on the cell membrane. These data support earlier evidence that cells with a more positive membrane potential will form pores more readily than cells with a more negative membrane potential; therefore, it is essential to find the cell membrane potential [19]. Cells have a different resting membrane potential based on cell type and status [20,21]. The cells’ membrane potential moves toward zero when active and working. However, when the cells are resting or quiescent, their membrane potential is more negative. The latent population of the HC69 cells, which is usually in a quiescent state, had a membrane potential that was more negative than the active version and uninfected parental C20 cells (Figure 2C). The parental cell line (C20) and HC69 active populations had no significant differences in their membrane potential. If HIV infection alters the membrane potential to move towards zero, then the similarity between C20 and the active population of HC69 cell membrane potential is the result of the C20 cells being a cell line and constantly proliferating. Hence, the membrane potential is already near zero. Comparing the membrane potential of primary HIV-infected microglia to uninfected ones would be more appropriate, but one limitation of this study is that obtaining primary microglia is difficult due to ethical issues. Another limitation of using the HC69 cells is that the HIV provirus lacks the HIV gag gene [14]. As mentioned, the gag gene produces HIV proteins: p17 (Matrix), p7 nucleocapsid, p24 (capsid), and p6. These HIV proteins are usually found at the host membrane, which can affect the membrane potential. Interestingly, when C20 cells are infected with the NL-AD8 virus, expressing all HIV viral proteins, the membrane potential is more depolarized than the control (Figure 3C). Other HIV proteins, including the viral protein U (Vpu) and Tat, have been shown to alter the cells’ resting membrane potential [22,23,24].

The method used to detect the membrane potential had some advantages and disadvantages. Measuring the membrane potential via flow cytometry can allow researchers to measure many cells rapidly, unlike the patch clamp method, in which only a few cells can be measured at a time [25,26,27]. Moreover, cell preparation was easy, only involving fixation and incubating the cells in a dye. This method is ideal for cells in suspension rather than adherent cells. It would be warranted to further compare this flow cytometry technique with the patch clamp method to ensure standardization. Nonetheless, we have shown that this method can be used to compare cell membrane potential rather than the absolute resting membrane potential.

To block and lock HIV transcription, the ZL0580 Tat inhibitor was implemented [13]. Although ZL0580 is marketed as a Tat inhibitor, it does not directly bind with or inhibit Tat. ZL0580 binds to bromodomain-containing protein 4 (BRD4), which is naturally found in human cells. Hence, ZL0580 might also affect other gene regulations, especially ones important for cell survival. Therefore, at high concentrations, ZL0580 can be toxic to cells. BRD4 is a protein from the bromodomain and extra terminal domain (BET) family, which regulates gene expression. BRD4 competes with Tat for P-TEFb/CDK9 interaction. When HIV Tat binds with P-TEFb, HIV elongation occurs; however, BRD4 binds with P-TEFb/CDK9 instead, which causes elongation to halt [28,29]. The ZL0580 dose–response curve showed that ZL0580 was toxic to HC69 microglial cells at 5 and 10 μM (Figure 4B). We showed that concentrations between 1 and 5 μM of ZL0580 worked best to suppress reactivation of HIV transcription.

To restrict HIV reactivation in latently infected cells, we explored the idea of using MMAE. MMAE disrupts microtubules and is highly toxic. MMAE is usually paired with antibodies to be less toxic and more effective [30]. We chose this drug because it is FDA-approved and has been used to treat brain cancers such as glioblastoma [31]. In addition, MMAE targets the microtubules. Microglial cells use many microtubules compared to other cells in the CNS, especially when the cells are transitioning from a resting state to an active state. In this study, we saw low cellular viability at 10 nM (Figure 4C). Considering this high toxicity, MMAE can cause peripheral and central neuropathy if free. To lower the potential of neuropathy, we paired MMAE with MENPs. Therefore, the potential use of this MMAE–MENP nanodrug would be less toxic and have the capacity to precisely target HIV-infected cells. Nanoparticles like MENPs can help reduce drug toxicity and precisely target the drug to the diseased tissues or cells [32].

As mentioned earlier, extracellular vesicles, specifically exosomes, are organic nanoparticles with potent drug-loading capacity, are non-immunogenic, and increase the drug half-life [33,34,35]. We used extracellular vesicles with our ZL0580. These extracellular vesicles have a hydrophobic lipid bilayer and an aqueous hydrophilic core that facilitate ZL0580 encapsulation. Studying extracellular vesicles uptake is warranted when designing a treatment plan. Extracellular vesicles labeled with the deep red plasma membrane (DRPM) dye were taken up rapidly within 10 min by the microglial cells, and after 30 min, most of the cells were positive for the dye (Figure 7). Interestingly, some cells seemed not to take up the stained extracellular vesicles. This could have occurred for various reasons, including cell location underneath other cells. However, over time, more labeled extracellular vesicles were taken up by the cells and cellular fluorescence was increased. This finding was like what other researchers found regarding EV cellular uptake [36,37,38]. The maximum uptake was seen at 24 h, suggesting that treatment with the ZLExos would be effective after 24 h.

Two loading methods, sonication and incubation, were implemented to load ZL0580 into extracellular vesicles (Figure 5A). Incubating and sonication were not significantly different from each other; both loaded about 80% of the ZL0580 drug. As mentioned earlier, the drug and extracellular vesicles are hydrophobic, meaning the interaction would have been instantaneous [39,40,41]. Sonication would have been more effective than incubation if the drug had been hydrophilic, as another researcher previously reported [42]. The hydrodynamic size of the ZLExos remained in the exosomal EV size range, which was confirmed with DLS and NTA. Also, as expected, the zeta potential of the ZLExos did not change significantly compared to the control because ZL0580 does not carry a charge.

For the next step in MELT, MMAE–MENPs were synthesized and characterized. MENP hydrodynamic size was measured via DLS and was estimated to be 193 nm. We understand that DLS is not the best method to measure the size of MENPs and that transmission electron microscopy (TEM) would have resulted in a smaller vesicle size. The nanoparticles may aggregate. Thus, the observed hydrodynamic size of the MENPs could be the result of the nanoparticle aggregation [43]. We were only able to access DLS as a resource for measuring particle size. For future studies, TEM will be incorporated.

The MENPs, surprisingly, were not toxic to the microglia cells even at higher concentrations. These results agree with other cell types, such as Hep2, HepG-2, SKMNC, and CT-26 [16,18,43,44]. MENP internalization by latently and actively infected microglial cells was examined. Since our previous data on the membrane potential indicated that the latent and active populations of HC69 cells have different membrane potentials, we assess MENP internalization into the active (GFP^Hi^) and latent (GFP^Lo^) populations. The internalization of MENPs is based on the cell membrane potential and the electrical pulse generated by the MENPs under a DC and AC magnetic field. Since the latent cell population has a membrane potential that is more negative than that of the active population, fewer MENPs will enter the cells than in the active population. These preliminary findings support this notion. The MENPs were labeled with oxonol III, and most entered the GFP^Hi^ population of the cells based on the high fluorescence upon removal of the background fluorescence (Figure 8B). In addition, increasing the magnetic field from 0.5 mT to 1.75 mT allowed more MENPs to enter the cells in both GFP^Hi^ and GFP^Lo^ populations. Time was also a significant factor in MENP internalization. Surprisingly, just incubating the HC69 cells for six hours with MENPs without an AC magnetic field increased the fluorescence of the cells, suggesting that these nanoparticles are taken up by microglia cells naturally. This suggests a potential need for coating to protect against non-specific uptake of the nanoparticles. Interestingly, the GFP^Lo^ cells had significantly more particles after 6 h, while the particle number in the GFP^Hi^ cells did not significantly change from the 30 min time point. This could mean that latent cell MENP internalization may be slower than that of actively infected cells. Lastly, methods such as cryo-EM, SEM, FTIR, and confocal microscopy could all be used to better characterize the nanoparticle/MELT internalization. Here we show that the two-step MELT process can potentially selectively target latent HIV-infected cells and can be developed as the first nanotherapeutic to treat HIV latency in the brain. Future in vitro and in vivo studies are needed to further investigate the potential use of tandem extracellular vesicles –MENPs to deliver drugs that specifically target HIV latency.

## 5. Conclusions

In summary, the use of MELT to target HIV latency shows promising results. First, we have shown that HIV infection modulates the membrane potential. Cells actively infected with HIV had a hyperpolarized membrane potential compared to uninfected and latently infected cells. This difference, a critical aspect of the MELT strategy, allowed MENPs to target latent (and active) HIV-infected cells and bypass uninfected cells precisely. This is the first report in which organic (extracellular vesicles) and inorganic (MENPs) nanoparticles coupled to ZL0580 and MMA (MELT) were used to target HIV infection in brain cells. Internalization of MELT by HC69 cells showed selective uptake of these nanoparticles and suppression of HIV reactivation. This is a proof-of-concept that tandem delivery of drugs within MELT nanoparticles can potentially target and treat latent HIV infection in the brain.

## Data Availability

The data presented in this study are available on request from the corresponding author due to university policy.
